# Metformin-Incorporated Gelatin/Nano-Hydroxyapatite Scaffolds Promotes Bone Regeneration in Critical Size Rat Alveolar Bone Defect Model

**DOI:** 10.3390/ijms23010558

**Published:** 2022-01-05

**Authors:** Chih-Hsiang Fang, Chung-Kai Sun, Yi-Wen Lin, Min-Chih Hung, Hung-Ying Lin, Ching-Hung Li, I-Ping Lin, Hung-Chen Chang, Jui-Sheng Sun, Jenny Zwei-Chieng Chang

**Affiliations:** 1 Trauma and Emergency Center, China Medical University Hospital, No. 2, Xueshi Rd., North Dist., Taichung City 40447, Taiwan; danny07291991@hotmail.com; 2 Institute of Traditional Medicine, School of Medicine, National Yang Ming Chiao Tung University, No. 155, Sec. 2, Linong Street, Taipei 11221, Taiwan; samcksun@gmail.com; 3 Institute of Biomedical Engineering, College of Medicine, National Taiwan University, No. 1, Sec. 4, Roosevelt Rd., Taipei 10617, Taiwan; zhew520@gmail.com; 4 Institute of Biomedical Engineering, College of Engineering, National Taiwan University, No. 1, Sec. 4, Roosevelt Rd., Taipei 10617, Taiwan; 5 Department of Dentistry, National Taiwan University Hospital, No. 1, Chang-Te Street, Taipei 10048, Taiwan; orthodaniel@yahoo.com.tw (M.-C.H.); ephedrine0626@hotmail.com (H.-Y.L.); r06422023@g.ntu.edu.tw (C.-H.L.); 6 School of Dentistry, College of Medicine, National Taiwan University, No. 1, Chang-Te Street, Taipei 10048, Taiwan; 7 Department of Dentistry, National Taiwan University Hospital, Hisnchu Branch, No. 25, Lane 442, Sec. 1, Jingguo Rd., Hsinchu City 30059, Taiwan; iping.lin1123@gmail.com; 8 Gin Chen Dental Clinic, No. 31, Long Chiang Rd., Taipei 10606, Taiwan; ben888chang@gmail.com; 9 College of Medicine, China Medical University, YingCai Campus, No. 91, Xueshi Rd., North Dist., Taichung City 40402, Taiwan; 10 College of Biomedical Engineering, China Medical University, YingCai Campus, No. 91, Xueshi Rd., North Dist., Taichung City 40402, Taiwan; 11 Department of Orthopedic Surgery, National Taiwan University Hospital, No. 7, Chung-Shan South Road, Taipei 10002, Taiwan

**Keywords:** metformin, nanocomposite, critical size defect, alveolar ridge preservation, bone regeneration

## Abstract

In this study, we fabricated gelatin/nano-hydroxyapatite/metformin scaffold (GHMS) and compared its effectiveness in bone regeneration with extraction-only, Sinbone, and Bio-Oss Collagen^®^ groups in a critical size rat alveolar bone defect model. GHMS was synthesized by co-precipitating calcium hydroxide and orthophosphoric acid within gelatin solution, incorporating metformin, and cross-linked by microbial transglutaminase. The morphology, characterization, and biocompatibility of scaffold were examined. The in vitro effects of GHMS on osteogenic gene and protein expressions were evaluated. In vivo bone formation was assessed in a critical size rat alveolar bone defect model with micro-computed tomography and histological examination by comparing GHMS with extraction-only, Sinbone, and Bio-Oss Collagen^®^. The synthesized GHMS had a highly interconnected porous structure with a mean pore size of 81.85 ± 13.8 µm. GHMS exhibited good biocompatibility; promoted ALPL, RUNX2, SP7, BGLAP, SPARC and Col1a1 gene expressions; and upregulated the synthesis of osteogenic proteins, including osteonectin, osteocalcin, and collagen type I. In critical size rat alveolar bone defects, GHMS showed superior bone regeneration compared to extraction-only, Sinbone, and Bio-Oss Collagen^®^ groups as manifested by greater alveolar ridge preservation, while more bone formation with a lower percentage of connective tissue and residual scaffold at the defect sites grafted with GHMS in histological staining. The GHMS presented in this study may be used as a potential bone substitute to regenerate alveolar bone. The good biocompatibility, relatively fast degradation, interconnected pores allowing vascularization, and higher bioactivity properties of the components of the GHMS (gelatin, nHA, and metformin) may contribute to direct osteogenesis.

## 1. Introduction

After loss of a natural tooth, the alveolar ridge undergoes resorption as a normal physiological process. The resorption is most rapid during the first year and may continue for more than 20 years. The continuing bone resorption poses difficulties in restoring the function and the esthetics of the extracted tooth with dental implants. To overcome the issue of bone loss, different materials, used in various combinations, have been grafted into the extraction socket to achieve alveolar ridge preservation (ARP). These include autogenous bone grafts, allografts, xenografts, and alloplasts that come in various forms, including particulate granules and sponges [1]. Although autogenous graft is considered the gold standard material for bone regeneration, it has inherent disadvantages, such as the need for a second surgical site to harvest bone grafts and marked resorption of the grafted bone. Allografts have the potential risks of immune response, infectious transmission of diseases, and low availability of bone banks [2,3]. Xenografts allow for unlimited supply of available material but may also present the risks of antigenicity and disease transmission. Because of the disadvantages of autografts, allografts, and xenografts, various alloplastic bone substitute materials have been applied to reduce post-extraction bone remodeling. At present, no conclusion can be made about which of the ARP techniques/biomaterials is the best [4,5]. There is no evidence that alloplastic materials render more favorable results than xenografts or allografts [1]. This has led to a need for research to focus on the development of better artificial bone substitutes.

Generally, the bone substitutes should closely mimic the compositions of the natural bone. Natural bone consists of mineral and organic components, i.e., hydroxyapatite (HA) and type I collagen. The collagen fibrils have a triple helical structure and serve as nucleation sites for HA deposition, but the low solubility of conventional HA grafts interferes with its degradation and replacement by new bone. By reducing HA particles to nanosize, the nano-hydroxyapatite (nHA) agglomerates may be easily taken up by cells and resorbed [6,7]. However, in comparison with bulk materials, nanoparticles exhibit high specific surface area and increased surface activities. Free nanoparticles/agglomerates are prone to adsorbing blood proteins, as well as to further phagocytosing and depositing in local tissue or disseminate systemically via blood circulation to activate inflammatory response or oxidative stress and induce toxicity [8]. Nanocomposites are a relatively new class of biocompatible materials that combine biopolymeric and biodegradable matrices with bioactive and easily resorbable nanoparticles [9]. In this study, gelatin, a hydrolytic form of collagen, was used in combination with nHA to mimic native bone tissue.

The most frequent causes of dental extractions prior to placement of an implant are linked to the presence of periodontal disease (68% to 69.20%) followed by endodontic failure (24.60% to 25.67%) [10]. Among those that presented endodontic failure previously, 23.38% to 50.41% subsequently developed peri-implantitis. Kim et al. reviewed 1226 tooth extraction records before implant placement and found 72.16%, 11.34%, and 2.12% of erratic extraction socket healing were due to periodontal pathology, combined periodontal-endodontic lesions, and endodontic pathology, respectively [11]. Therefore, for the benefit of implant integration, potential implant sites with previous infection should be managed prior to implant placement. It has already been well documented that there is a close association between periodontal disease and diabetes mellitus (DM). A recent literature review has shown that DM is also associated with a higher prevalence of periapical lesions, greater size of osteolytic lesions, greater likelihood of asymptomatic infections, and worse prognosis for root-filled teeth [12]. Metformin, 1,1-dimethylbiguanide, is currently recommended as the first-line therapy for type 2 DM. Metformin not only decreases hyperglycemia and insulin resistance but also has been shown to have potential anti-inflammatory [13], anticancer, and neuroprotective [14] effects, cardiovascular benefits [15,16], as well as to improve bone metabolism [17]. Diabetic patients can develop diabetic osteopathy, including osteopenia, osteoporosis, and an increased incidence of low stress fractures [17]. The use of metformin appears to have a good safety profile regarding the bone [17,18].

Most in vitro studies have pointed to metformin having a bone-anabolic effect: tipping the phenotypic balance of bone marrow stromal cells towards osteoblastogenesis, increasing the bone-forming capacity of osteoblasts, and decreasing the recruitment and bone-resorbing activity of osteoclasts [17]. In addition, metformin inhibits formation of reactive oxygen species (ROS) and apoptosis in osteoblastic cultures exposed to high glucose concentrations [19] or advanced glycation end products (AGEs) [20]. In a rat model of ligature-induced periodontitis, metformin treatment induced a significant reduction in alveolar bone loss [21]. Randomized controlled clinical trials have shown that local delivery of metformin gel into the periodontal pocket or intrabony defects results in a significant increase in probing depth reduction, clinical attachment level gain, and defect improvement in patients with chronic periodontitis [22,23,24]. In a rat model of induced periapical lesions, administration of metformin systemically by daily intramuscular injections inhibits the progression of periapical lesion [25]. Our previous studies further show that application of metformin as an intracanal medication ameliorates periapical lesions through suppression of hypoxia-induced apoptosis of osteoblasts [26] and suppression of monocyte recruitment via modulation of inducible nitric oxide synthase expression and nitric oxide production [27] in rats. Given that osteoblasts can uptake metformin directly [28], and that previous studies support the therapeutic benefits of local metformin application in periodontal and periapical diseases, we speculate that incorporating metformin into gelatin/nHA nanocomposite may promote bone regeneration in alveolar bone defects.

Bio-Oss^®^, a deproteinized bovine bone mineral, is one of the most widely used biomaterials in alveolar ridge preservation procedures. This osteoconductive graft has a trabecular and porous structure similar to human bone and acts as a scaffold for host bone ingrowth. However, Bio-Oss^®^ degrades slowly and may induce fibrous encapsulation with healing [29]. As an alternative, Bio-Oss Collagen^®^, a composite of 90% Bio-Oss^®^ spongiosa granules embedded in a 10% biodegradable porcine-derived collagen type I, has been introduced. According to the manufacturer, the addition of collagen to the mineral matrix acts as a cohesive for the Bio-Oss^®^ particles, which makes Bio-Oss Collagen^®^ formable and allows a good adaptation and stabilization of the graft to the morphology of a defect. Sinbone consists of 60% hydroxyapatite (HA) and 40% β-tricalcium phosphate (β-TCP). Theoretically, β-TCP undergoes biologic degradation 10 to 20 times faster than HA but is mechanically weaker [3]. The combination of the two may offer both advantages clinically. In this study, the performance of gelatin/nHA/metformin scaffold (GHMS) in the form of sponge to promote alveolar bone regeneration in a critical size rat alveolar bone defect model was evaluated in comparison with commercially available xenograft (Bio-Oss Collagen^®^) and alloplast (Sinbone granules).

## 2. Results

### 2.1. Characterization of Gelatin/Nano-Hydroxyapatite/Metformin Scaffold (GHMS)

#### 2.1.1. Morphology of GHMS

**Figure 1:**
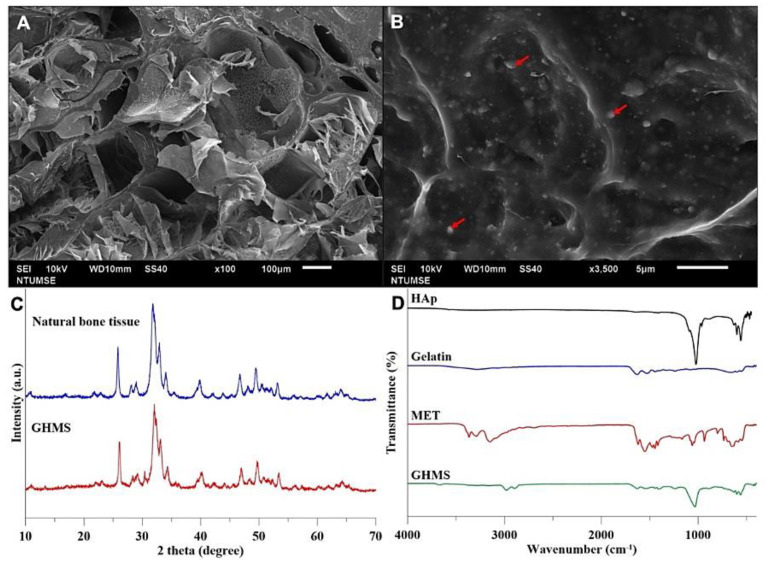
Characterization of Gelatin/Nano-Hydroxyapatite/Metformin scaffold (GHMS). Scanning electron microscopy (SEM) images of GHMS at different magnifications: (**A**) 100× and (**B**) 3500× (right). (**C**) X-ray diffractometry (XRD) patterns of natural bone tissue and GHMS. (**D**) Fourier transform infrared (FTIR) patterns of conventional hydroxyapatite (HAp), gelatin, metformin (MET), and GHMS (Table 1).

The SEM image show the synthesized scaffold had highly interconnected porous structure with a mean pore size of 81.85 ± 13.8 µm (Figure 1A,B). The images show that the GHMS has an open and interconnected porous structure. Red arrows indicate metformin embedded into the structure of GHMS.

#### 2.1.2. Crystal Phase Identification

The XRD pattern of the HA in GHMS was similar to the crystalline phase of natural bone (Figure 1C), showing predominant peaks matching those of the conventional HA. The GHMS demonstrated broad diffractions corresponding to (002), (211), (300), (202), (130), (002), (222), and (213) of the conventional hydroxyapatite. The results confirmed the formation of hydroxyapatite mineralization.

#### 2.1.3. Functional Group Identification

The FTIR spectra of the GHMS confirmed the presence of the characteristic adsorption bands of gelatin, HA, and metformin (Figure 1D). The characteristic peaks for HAp were located in the 500–1100 cm^−1^ region. The asymmetric bending and the stretching band of the (PO4)3- group was found at 1063 cm^−1^. In addition, characteristic peaks for gelatin were observed at 2800–2950 cm^−1^ (C-H stretching), 1652 cm^−1^ (C = O group), and 3420 cm^−1^ (O-H stretching), respectively. Two typical bands at 3369 cm^−1^ and 3294 cm^−1^, relative to the N-H primary stretching vibration, a band at 3155 cm^−1^ due to the N-H secondary stretching, and characteristic bands at 1626 cm^−1^ and 1567 cm^−1^ assigned to C-N stretching were observed in the FTIR spectrum of pure MET.

### 2.2. Biocompatibility of Gelatin/Nano-Hydroxyapatite/Metformin Scaffold (GHMS)

**Figure 2:**
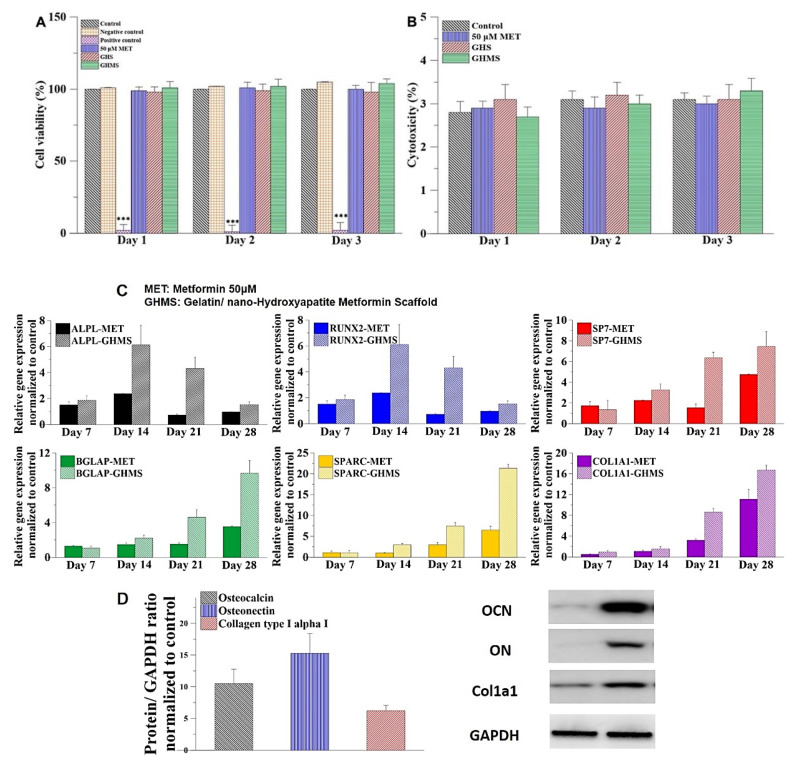
In vitro studies to evaluate the biocompatibility and osteogenic potentials of gelatin/nano-hydroxyapatite/metformin scaffold (GHMS). (**A**) Water-soluble tetrazolium (WST-1) assay. (*n* = 12; *** *p* < 0.001) (**B**) Extracellular lactate dehydrogenase (LDH) Assay. (**C**) Osteogenetic gene expression and protein synthesis after coculturing human mesenchymal stem cells (hMSCs) with GHMS or 50 μM metformin (normalized to the control group). The osteoblast-specific genes of the hMSCs, including alkaline phosphatase (ALPL), runt-related transcription factor-2 (RUNX2), osterix (SP7), osteocalcin (BGLAP), osteonectin (SPARC), and type I collagen (COL1A1) were upregulated compared to the control group. (**D**) Osteogenetic protein synthesis after co-culturing hMSCs with GHMS. The hMSCs co-cultured with the GHMS for four weeks showed osteogenic protein overexpression of osteocalcin (OCN), osteonectin (ON), and collagen type I (Col1a1) compared to the control group.

The cell viability was evaluated on L929 cell line for three days comparing 0.2 g/mL of the GHMS sample with blank control, negative control (aluminum oxide), positive control (zinc diethyldithiocarbamate), 50 μM metformin, and gelatin/nano-hydroxyapatite nanocomposite scaffold (GHS; no metformin). Results of the WST-1 assays showed no significant differences in the L929 fibroblast viability when cultured with negative control, 50 μM metformin, GHS, and GHMS compared with blank control (Figure 2A), indicating the cells were able to grow and proliferate well in the presence of GHMS. The cytotoxicity was evaluated by determination of LDH release following treatment of L929 cell line with 0.2 g/mL GHMS, GHS, 50 μM metformin, or no treatment. The results of the LDH assays showed no significant differences between blank control and the experimental groups (Figure 2B), with cytotoxicity less than 3.5%. Based on the standards of ISO-10993, GHMS exhibited good biocompatibility.

### 2.3. Osteogenic Genes Expression and Protein Synthesis

The qRT-PCR analysis revealed that osteogenic genes of the GHMS-treated hMSCs, including ALPL, RUNX2, SP7, BGLAP, SPARC, and Col1A1, were more upregulated compared to the control or metformin treatment alone (Figure 2C). Western blotting also showed significant upregulation of osteogenic proteins, including osteonectin, osteocalcin, and collagen type I after coculturing hMSCs with GHMS for four weeks (Figure 2D). These results indicated that the GHMS promoted in vitro osteogenesis in the hMSCs.

### 2.4. Micro-Computed Tomography (Micro-CT) Analysis

**Figure 3:**
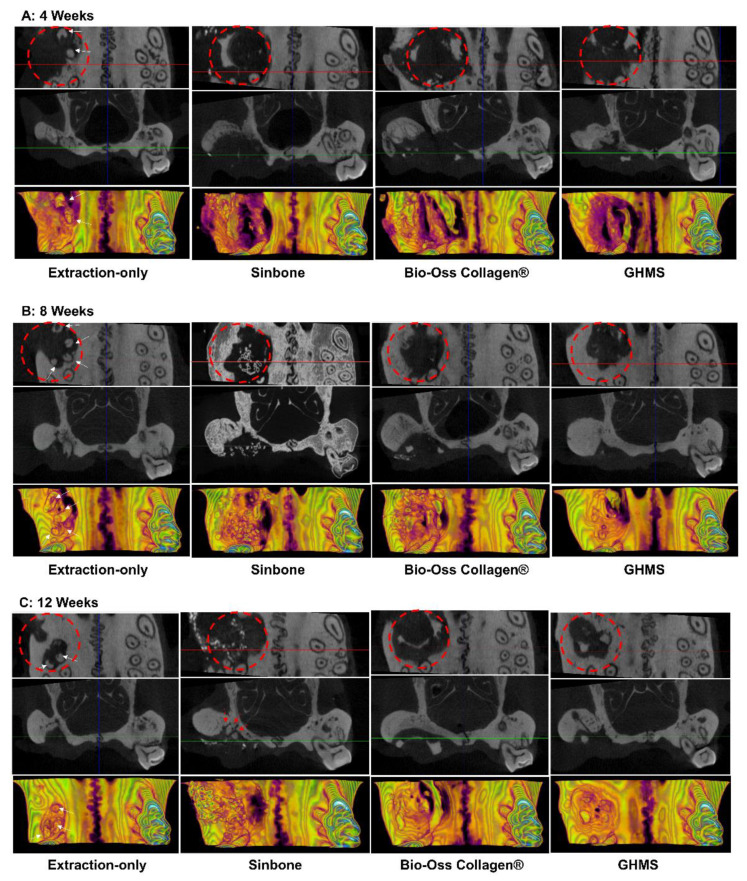

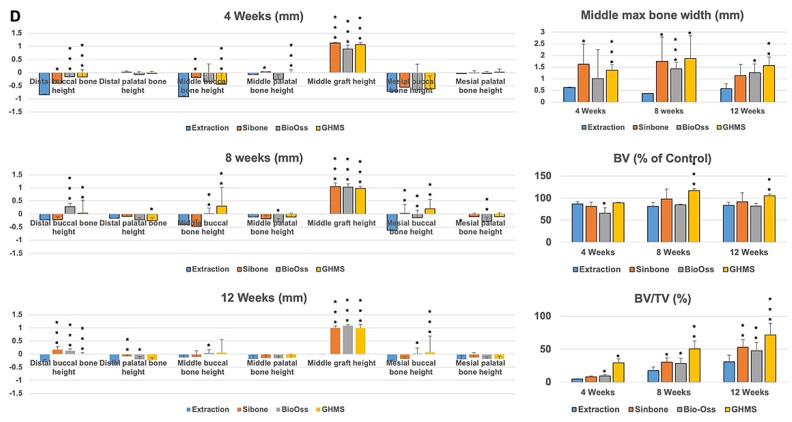
Comparison of the effectiveness of alveolar ridge bone regeneration at maxillary first molar (M1) examined by micro-computed tomography (micro-CT). Representative images show the coronal (top) and transaxial (middle) sections in the middle part of M1 site and the 3D occlusal view (bottom) of the extraction-only (without creating bone defect), Sinbone, Bio-Oss Collagen^®^, and gelatin/nano-hydroxyapatite/metformin (GHMS) groups at (**A**) 4, (**B**) 8, and (**C**) 12 weeks. Please note that some root fragments remained in the extraction-only groups (white arrow). Substantial amount of residual grafting material at the defect site in the Sinbone group was evident after 12 weeks. (**D**) Analysis of bone heights, middle maximum bone width, bone volume (BV% of control), and bone fill (percentage of bone volume/total volume; BV/TV%) at different time points. (*: *p* < 0.05; **: *p* < 0.01; ***: *p* < 0.001).

Representatives of the 3D reconstructed and sectioned micro-CT images at bilateral maxillary first molar regions for the four different groups (extraction-only, Sinbone granules, Bio-Oss Collagen^®^, and GHMS) collected at 4, 8, and 12 weeks are shown in Figure 3A–C. The grafting groups had fuller buccal bone contour and better bone height compared to the extraction-only group. From gross inspection, the extraction group exhibited comparative bone width to but substantially less bone height than the contralateral non-extraction side, while all grafting groups showed considerably larger bone width on the experimental side. Twelve weeks after grafting, the defect sites still presented with significant amount of Sinbone granules and Bio-Oss Collagen^®^, indicating the slow resorbing nature of these materials, whilst most of the GHMS material had already degraded. The GHMS group showed better new bone regeneration inside the socket/defect (alongside the periphery of the socket/defect underneath the graft material) compared with other groups.

Results of the micro-CT measurements of bone heights, widths, volumes, and extents of bone fill at 4, 8, and 12 weeks are shown in Figure 3D. Bone heights were measured at the buccal and palatal aspects in the distal, middle, and mesial transaxial sections of the M1 site and presented as the height difference between the experimental and non-extraction sides of the same rat by subtracting the corresponding bone height of the non-extraction side from the height on the experimental side (a positive measurement indicating gain in bone height relative to the non-extraction side). Graft heights were measured in grafting groups in only the middle section of the M1 site and presented by subtracting the middle palatal bone height of the non-extraction side from the graft height measurement. Maximum bone width was measured in the middle section of the M1 site and presented as the width difference between the experimental and non-extraction sides by subtracting the corresponding bone width on the non-extraction side from the width on the experimental side (a positive measurement indicating gain in bone width relative to the non-extraction side). Bone volumes were measured on the experimental side by adding up consecutive bone surfaces excluding the graft material on the transaxial sections mesiodistally throughout the M1 site and presented as percentage (of control) by dividing from the bone contour volume on the non-extraction side. Bone fill was measured as bone volume density or bone volume/total volume (BV/TV%) in the predefined ROI to indicate the percentage of bone present inside the defect. Generally, the grafting groups preserved the buccal and palatal bone heights. At 4 weeks, all grafting groups showed some loss in the buccal bone heights. By 8 and 12 weeks, buccal bone heights mostly improved. The graft heights in the middle section of M1 were greater than the palatal height of the contralateral non-extraction side and maintained throughout the experimental periods. There was a significantly greater increase in bone width for the grafting groups than the extraction-only group. The GHMS group exhibited significantly greater bone volume than other groups at 8 and 12 weeks. During the postoperative period, percentage of bone fill (BV/TV%) inside the defect increased between 4 to 12 weeks. The GHMS group was overall the best to promote new bone fill.

### 2.5. Histological Analysis

**Figure 4:**
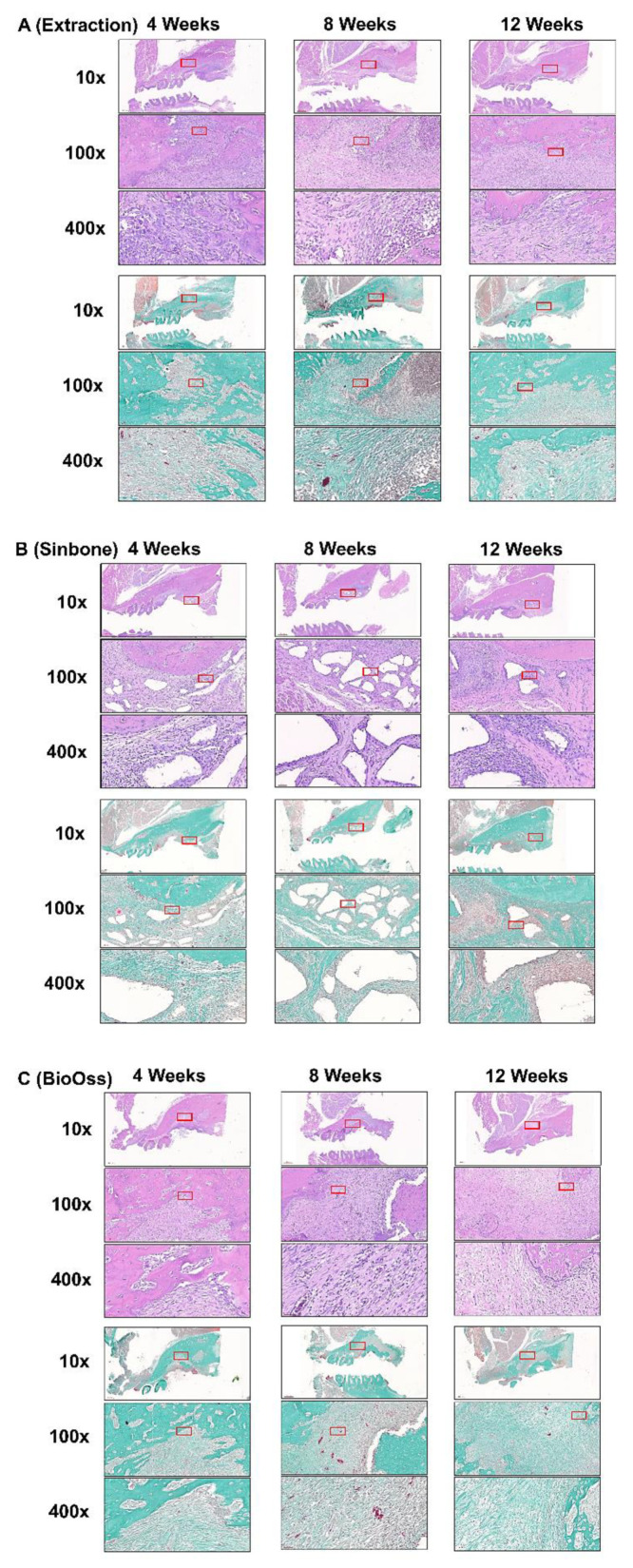

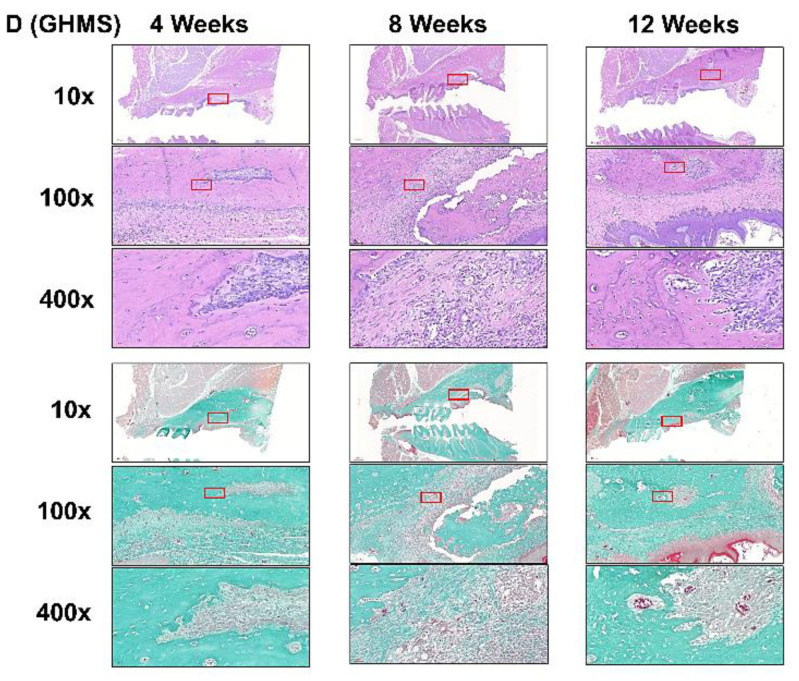
Representative histological sections of the maxillary first molar (M1) to third molar (M3) region of rats after M1 (**A**) extraction or grafting with (**B**) Sinbone, (**C**) Bio-Oss Collagen^®^, or (**D**) gelatin/nano-hydroxyapatite/metformin (GHMS) by hematoxylin and eosin (H&E) and Goldner’s Trichrome staining at 4, 8, and 12 weeks. At the 12th week after extraction, new bone growth was less evident in the extraction-only group. The defects grafted with Sinbone granules showed a substantial amount of graft material remained in the defect site. Bio-Oss Collagen^®^ showed some bone regeneration in the defect site. In contrast, well-developed new bone and the presence of haversian canals were found in the GHMS sections. In addition, there was no detectable fibrous tissue invasion or inflammatory cells infiltration in the GHMS experimental group. Goldner’s trichrome staining shows significantly less new bone formation in the extraction-only group, whereas immature bone formation and soft tissue invasion were observed in Sinbone. In the GHMS samples, there was a well-developed new bone, and angiogenesis were found. The red box is the position of magnification.

Representative histological images of the extraction-only, Sinbone, Bio-Oss Collagen^®^, and GHMS specimens by H&E and Goldner’s trichrome staining are shown in Figure 4A–D. All the experimental groups showed a certain extent of bone formation within the extraction/defect site, mostly in the apical portion of the socket/defect. At the 4th week, the extraction-only specimens exhibited richly vascularized and heavy infiltrated disorganized tissue in the socket, whereas by the 12th week, organization of the extracellular matrix collagen was evident and the collagen fibers were arrayed in a more parallel fashion (Figure 4A). During the grafting procedure, Sinbone granules were easily crushed and condensed into the defect site. These granules were present not only in close proximity to the defect walls but also in locations outside the defect (Figure 4B). At the 4th week, substantial amount of Sinbone particles were encapsulated by loose connective tissue infiltrated with polymorphonuclear and mononuclear cells. By the 12th week, many of the Sinbone granules did not degrade and remained within the defect. Bio-Oss Collagen^®^ specimens showed some bone regeneration in the apical part of the defect, but the central portion was mostly occupied by densely packed connective tissue fibers and mesenchymal cells (Figure 4C). By the 12th week after Bio-Oss Collagen^®^ grafting, the extent of new bone regeneration or mineralization was still unsatisfactory. In contrast, active formation of new bone with the presence of Harversian canals was observed in the GHMS group in both the apical part and along the lateral walls of the defect (Figure 4D). There was no detectable fibrous tissue invasion or inflammatory cells infiltration. By the 12th week, most the GHMS had already degraded, while large amounts of woven bone had almost filled the defect.

## 3. Discussion

**Figure 5:**
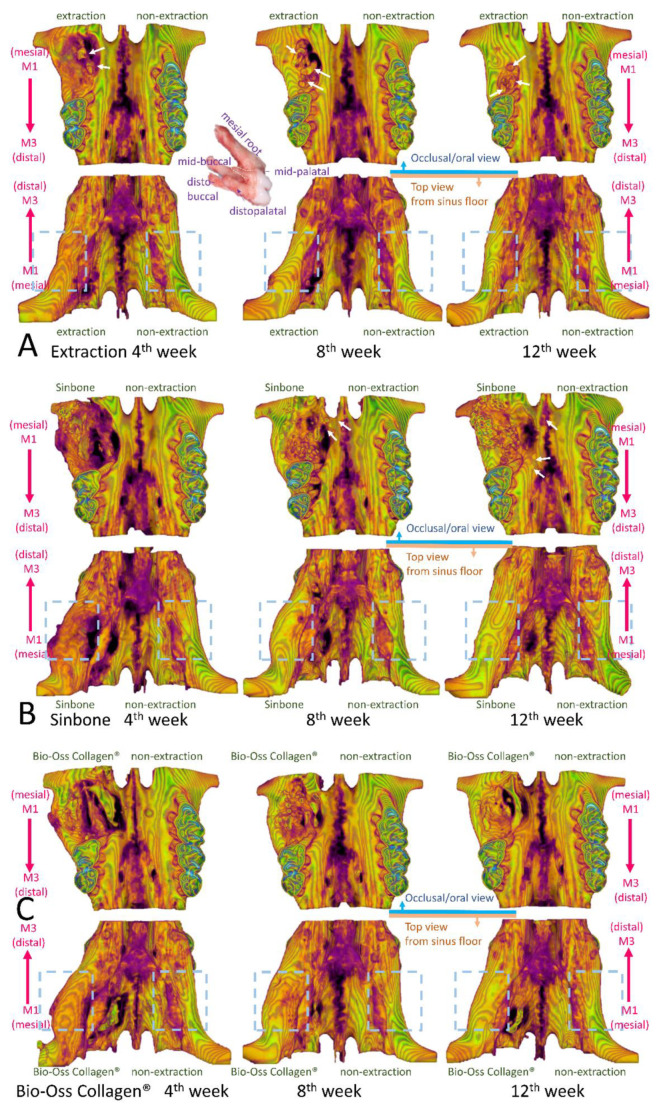

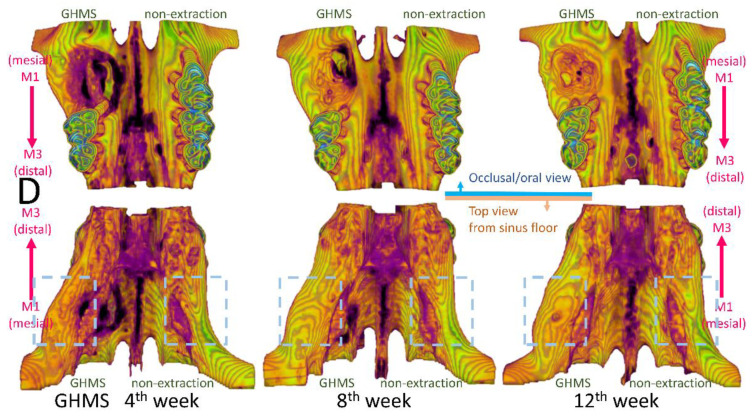
Representative three-dimensional micro-computed tomography (micro-CT) images of the maxillary first molar (M1) to third molar (M3) region of rats viewing from the occlusal/oral side and the top/sinus floor side after M1 (**A**) extraction or grafting with (**B**) Sinbone, (**C**) Bio-Oss Collagen^®^, or (**D**) gelatin/nano-hydroxyapatite/metformin (GHMS) at the 4th, 8th, and 12th weeks.

Any new bone substitute should be tested both in vivo and in vitro prior to testing in humans. To evaluate GHMS in a standardized and discriminative manner, we have adopted a simple yet cost-effective critical size rat alveolar defect model. Prior to conducting this study, we tested the protocols with additional rats in a pilot study. Our protocols included decoronation of the maxillary M1 two weeks before creating the alveolar bone defect. This preceding decoronation procedure facilitated complete and atraumatic removal of the five roots, as otherwise M1 roots (white arrows in Figure 3), especially the mid-palatal root, (Figure 5A) could easily have fractured during extraction. Decoronation also preserved greater hard and soft tissue envelopes/dimensions for accommodation of a greater amount of grafting materials and tension-free primary closure. Results from our pilot study showed that by using a 2.7 mm diameter bur in creating bone defect to the depth that the bur was just submerged into the soft tissue surface, we would create an approximately 3 mm diameter and 2 mm depth critical size defect (CSD) in the alveolar bone of eight-week-old rat. With this rat model, our new biomaterial (GHMS) has demonstrated better performance than the commercially available xenograft (Bio-Oss Collagen^®^) and alloplast (Sinbone). Although all three grafting materials showed comparable ability to preserve ridge dimensions (in heights and widths), GHMS presented significantly greater bone volume gain (BV% of control) and more bone fill (BV/TV%) than others.

Results from meta-analyses show that ARP preserves 1.31–1.54 mm bucco-oral bone width and 0.91–1.12 mm bone height in comparison with unassisted socket healing [5]. However, most of the studies in this meta-analysis have evaluated only single-rooted teeth. In a study that investigated the effects of ARP using Bio-Oss Collagen^®^ plus collagen membrane in periodontally compromised maxillary molar sockets [30], the results showed no significant differences for the central and palatal alveolar ridge measurements between treated and control groups. In another study that evaluated preservation of periodontally involved molar sockets with Bio-Oss^®^ particles plus collagen membrane [31], the results also showed no significant vertical differences in the medium region of the sockets between the experimental and natural healing groups. Therefore, for long-span extraction spaces such as the molar region, the preservation of vertical height in the mid-central part of the edentulous ridge may be suboptimal.

In this study, we showed that all grafting groups preserved bone height, especially at the buccal side, whereas the unassisted extraction group showed considerable loss of buccal bone height. Our study’s grafting groups also showed extreme bone width gain in comparison with the non-extraction control side. The bone volumes (excluding graft material) of GHMS group at 8 and 12 weeks were even greater than the non-extraction contralateral side (BV% of control >100%), indicating that GHMS regenerated bone beyond existing bone limits. This was probably due to the CSD model that we used. In this rat model, we created an extremely large defect in the grafting groups. To a certain extent, this was similar to the clinical scenario of performing alveolar ridge split technique for horizontal bone augmentation [32,33]. The serial transaxial sections on micro-CT clearly showed that the alveolar ridge of the defect/grafting side at M1 bended gradually more outward buccally and upward apically from the most distal section (image at upper left corner) to the mesial section (image at lower right corner). The bone width at the mesial site of M1 appeared to increase significantly more than that at the distal side (blue dashed box in Figure 5). Therefore, even though the defect did not completely fill with new bone, the overall bone volume became larger than the non-extraction side.

Since M1 has divergent roots and a very thin and slender mid-palatal root (yellow dashed line), the roots are prone to fracture during extraction (white arrows in Figure 5A). The buccal contour at the extraction site was more concave than the grafted sites in (B–D) due to higher resorption. The alveolar ridge width from top view appeared to be similar to the non-extraction side (blue dashed box) in the extraction group. (B–D) The buccal contours at the grafted sites were convex. The alveolar ridge width appeared to be substantially wider at the mesial side of the M1 grafted site when compared to the non-extraction side, especially when viewing from the top (blue dashed box). Some Sinbone granules were located outside the defect site (white arrows in Figure 5B). The Sinbone granules blended with the native bone, and it was sometimes difficult to distinguish them from native bone on micro-CT.

Bio-Oss Collagen^®^ grafting to preserve the alveolar ridge has been evaluated in experimental animal models and clinical human settings with varying success. Results from animal studies show that although Bio-Oss Collagen^®^ preserves alveolar ridge dimension, it may in fact delay healing due to slow resorption of the material [34,35]. These results were based on a dog model with a relatively small bone defect, but the results are consistent with our CSD rat model. Although clinical studies have concluded a positive role of Bio-Oss Collagen^®^ in ridge preservation, these studies have evaluated Bio-Oss Collagen^®^ grafting with a collagen membrane or matrix. The likely benefit from the barrier membrane or collagen matrix is overlooked. Therefore, the clinical treatment effects from solely Bio-Oss Collagen^®^ grafting are still inconclusive. Sinbone comprises 60% HA and 40% β-TCP. In theory, it should be rapidly replaced by regenerative bone because of the β-TCP content. However, our study showed that a considerable amount of Sinbone granules at the defect site remained unresorbed at the 12th week. Some of these granules were present in locations outside the defect (white arrows in Figure 5B), while most of the GHMS material had already degraded (Figure 5D).

Although Sinbone seemed to perform better than Bio-Oss Collagen^®^ in terms of bone volume and bone fill, these results should be interpreted with care. Because the condensed Sinbone granules were in close vicinity to the defect walls and the mineral content of Sinbone was similar to the surrounding bone, there were difficulties in distinguishing between Sinbone and native bone (red arrows in Figure 3C and white arrows in Figure 5B) in micro-CT sections/images [36]. Thus, the micro-CT measurement of Sinbone group may be overestimated. Nevertheless, GHMS still exhibited superior bone regeneration capacity compared with Sinbone when evaluated with micro-CT.

An ideal bone graft material requires osteogenic, osteoinductive, and osteoconductive properties. Xenografts and alloplasts generally show only osteoconductive properties [1]. An ideal bone substitute should also be biocompatible, biodegradable (concordant with simultaneous normal bone replacement), microporous (for angiogenesis and bone ingrowth), of sufficient mechanical strength (to prevent wound from collapsing), and flexible to easily fit the defect shape. In this study, we fabricated GHMS in sponge form to facilitate handling. Micro-CT and histologic assessments verified that GHMS biodegraded concomitantly with new bone regeneration. The enhancement of osteogenesis partly attributes to the nano-size of HA. In contrast to micron-size HA, nHA mediates M2 macrophage polarization, which enhances mesenchymal stem cell osteogenesis in vitro and promotes tissue vascularization and increases bone volume in vivo [37]. The nHA bone graft in combination with the collagen membrane has resulted in significant probing pocket depth reduction, clinical attachment level gain, and increase of radiographic bone fill in comparison with open-flap debridement alone [38]. Despite the potential of nHA particles for medical and dental applications, controversies about their safety exist. In glioma cells, nHA increases reactive oxygen species (ROS) production, induces DNA damages, exerts cytotoxic effects, and prevents cellular proliferation and migration [39]. By incorporating nHA fillers into gelatin, we fabricated non-toxic biomimetic nanocomposite biomaterial. WST-1 and LDH assays confirmed that GHMS had met the ISO-10993 standards for biocompatibility.

Gelatin has more biological functional groups and is widely used in pharmaceuticals due to its good cell viability, lack of antigenicity, shape availability, and cost efficiency. However, the application of gelatin in bone regeneration is limited by its rapid degradation and lack of mechanical strength. To improve its mechanical strength, we used transglutaminase to crosslink gelatin instead of using glutaraldehide, which may present concerns regarding cytotoxicity [40]. Previous studies have suggested a minimum pore size of 100 μm for bone regeneration scaffold [41], while the upper limit in porosity and pore size of the scaffold is constrained by mechanical requirements. By adjusting the concentration of both gelatin and nHA to 10% w/v, the compressive strength of our gelatin/nHA nanocomposite is similar to that of cancellous bone. The resultant GHMS showed sufficient mechanical strength to prevent soft tissue collapsing as evident from the sustained graft height seen at the 12th week (Figure 3D). SEM showed that GHMS is a sponge-like macroporous scaffold with an average pore size of 81 μm and a highly interconnected porous structure (Figure 1A), which may have allowed better oxygen and nutrient distribution, cell migration and angiogenesis through the scaffold.

Although most bone graft substitutes do not show osteoinductive properties, Kim et al. demonstrated that human osteoblastic cells prefer to attach onto gelatin/nHA nanocomposites than onto conventional gelatin/HA composites to proliferate and secrete alkaline phosphatase (ALP) and osteocalcin [42]. Gelatin hydrogel may serve as a release carrier of growth factors to promote bone regeneration [43]. Our previous study has shown that biomimetic gelatin/nHA microspheres may control release of stromal cell-derived factor-1 proteins to attract the migration of stem cells in vitro and enhance alveolar bone regeneration in vivo [44]. Several studies have reported the potential osteogenic effect of metformin in osteoblastic cell differentiation, bone matrix synthesis, and osteoblast proliferation by activation of the 5′ adenosine monophosphate (AMP)-activated protein kinase (AMPK) [45]. Metformin has a positive effect on osteoblast differentiation due to increased activity of RUNX2 via AMPK/upstream stimulatory factor-1/small heterodimer partner regulatory cascade. Metformin significantly stimulated ALP activity, enhanced mineralized nodule formation, and increased expression of RUNX2 and osterix in induced pluripotent stem cell–derived MSCs partly via the LKB1/AMPK pathway [46]. In this study, metformin alone exhibited osteoinductive potential by upregulating the expressions of ALPL, RUNX2, SP7, BGLAP, SPARC, and COL1A1 genes, while GMHS upregulated these osteogenic genes even more (Figure 2C), indicating that synergism of gelatin/nHA scaffold and metformin improved the osteoinductivity of the materials and further increased osteogenesis. Considering that metformin is an effective drug for osteogenic differentiation of MSCs into osteogenic lineages and that its anti-inflammatory potential shows therapeutic benefits in periodontal and periapical diseases, future research may involve application of GHMS in chronically infected alveolar socket/defect models. Sustained delivery of metformin may be designed into future prototype bone tissue scaffolds to promote bone healing in large bone defects.

## 4. Materials and Methods

### 4.1. Materials

Calcium hydroxide (Ca(OH)2, Cas. No. 1305-62-0), gelatin (Cas. No. 9000-70-8), hydrochloric acid (HCl, Cas. No. 7647-01-0), and metformin (Cas. No. 1115-70-4) were purchased from Sigma-Aldrich (St. Louis, MO, USA). Orthophosphoric acid (H3PO4, Cas. No. 7664-38-2), sodium hydroxide (NaOH, Cas. No. 1310-73-2), and microbial transglutaminase (MTGase) were acquired from T. Baker (Center Valley, PA, USA), SHOWA (Meguro, Tokyo, Japan), and Activa (Ajinomoto, Japan), respectively.

### 4.2. Fabrication of Gelatin/Nano-Hydroxyapatite/Metformin Scaffold (GHMS)

The gelatin/nHA/metformin nanocomposite scaffold/sponge was fabricated as previously described with some modification by coprecipitating HA within gelatin solution to the weight ratio of 1:1, adding 50μM metformin, cross-linked by 0.1% MTGase, and freeze-drying in 24 well plate. [44].

### 4.3. Characterization of Gelatin/Nano-Hydroxyapatite/Metformin Scaffold (GHMS)

The gelatin/nHA/metformin scaffold (GHMS) specimens were gold sputtered and viewed with Hitachi S3000/N scanning electron microscope (Hitachi, Ltd., Tokyo, Japan). The crystal phase of GHMS was evaluated using X-ray diffractometer (Rigaku Corporation, The Woodlands, TX, USA), and the obtained patterns were analyzed with a model auto-matched to the International Center for Diffraction (ICDD) database using Jade 6.0 software (Materials Data Inc., Livermore, CA, USA). Fourier transform infrared (FTIR) spectroscopy was performed to determine the chemical groups and the crystalline mineral phases present in GHMS comparing with pure gelatin, pure HA, and metformin. The spectra were recorded at wavelengths in the range of 450 to 4000 cm^−1^.

### 4.4. In Vitro Study

#### 4.4.1. Isolation and Culture of Human Mesenchymal Stem Cells (hMSCs)

Following an approved Institutional Review Board protocol from the National Taiwan University Hospital (NTUH IRB No. 201,704,005 RINA), hMSCs were collected from bone marrow aspiration during total hip or knee joint replacement surgery and mononuclear cell fraction was isolated using Ficoll-Paque PLUS (GE Healthcare, Amersham, UK). In subsequent experiments, the hMSCs were used at passages 3–4.

#### 4.4.2. Cell Viability of Gelatin/Nano-Hydroxyapatite/Metformin Scaffold (GHMS)

The biocompatibility of GHMS was evaluated in accordance with ISO 10993-5 by water-soluble tetrazolium (WST-1) assay on L929 cell line (Bioresource Collection and Research Center, Hsinchu, Taiwan) comparing 0.2 g/mL of the GHMS sample with blank control, negative control (aluminum oxide; Sigma-Aldrich), positive control (zinc diethyldithiocarbamate; Sigma-Aldrich, St. Louis, MO, USA), 50μM metformin, and gelatin/nHA nanocomposite scaffold (GHS; no metformin) [44,47]. The cell viability was calculated using the following formula:

(1) $$\mathrm{Cell}\;\mathrm{viability}\;\left(\%\right)=\frac{\left(\mathrm{OD}\;\mathrm{experiment}\;-\;\mathrm{OD}\;\mathrm{background}\right)\times 100}{\left(\mathrm{OD}\;\mathrm{control}\;-\;\mathrm{OD}\;\mathrm{background}\right)}$$

#### 4.4.3. Cytotoxicity

Extracellular lactate dehydrogenase (LDH) release following treatment of L929 cell line with 0.2 g/mL GHMS, GHS, 50 μM metformin, or no treatment was determined using a CytoTox 96 Assay Kit (Promega Corporation, Madison, WI, USA) to evaluate possible toxicity.

#### 4.4.4. Gene Expression

Quantitative real-time polymerase chain reaction (qRT-PCR) was used to investigate the relative expression fold changes of alkaline phosphatase (ALPP), runt-related transcription factor 2 (RUNX2), osterix (SP7), osteocalcin (OCN or BGLAP), osteonectin (ON or SPARC), and collagen type 1 (COL1Al) at days 7, 14, 21, and 28 after seeding hMSCs with GHMS or 50 μM metformin using glyceraldehyde 3-phosphate dehydrogenase (GAPDH) as internal control [44,47].

#### 4.4.5. Protein Expression

Isolation of osteocalcin (OCN), osteonectin (ON), and collagen type I (Col1a1) proteins from the QIAzol-lysed GHMS samples were performed and assessed by western blotting as previously described [44]. The metformin-free drug was used as the control group and using glyceraldehyde 3-phosphate dehydrogenase (GAPDH) as internal control.

### 4.5. In Vivo Study

**Figure 6:**
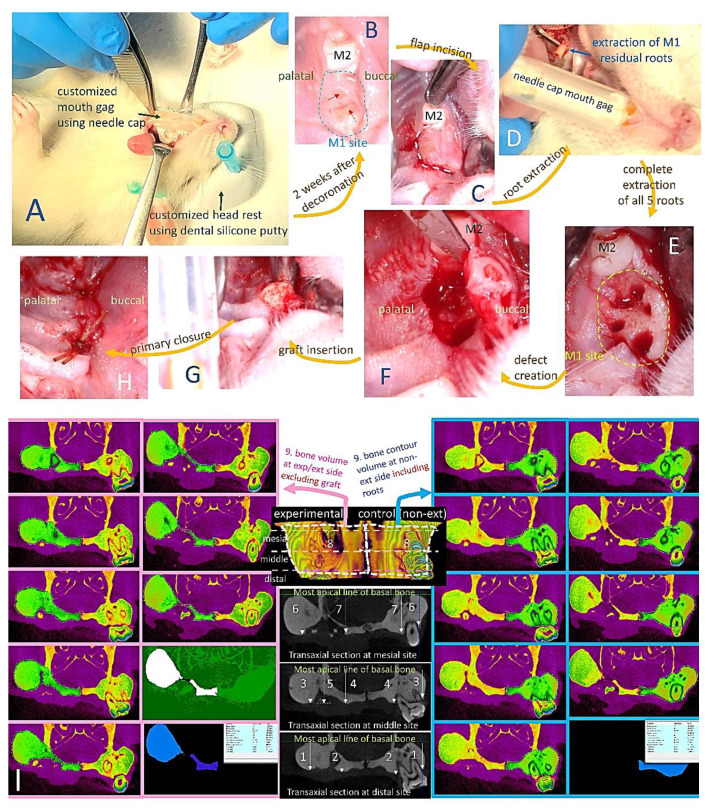
The experimental procedures of the critical size rat alveolar bone defect model and measurements of parameters. (**A**) Rat was positioned in dorsal recumbency with customized mouth gag (using needle cap) and head rest (fabricated with dental silicone putty). On the experimental/grafting side, decoronation of maxillary first molar (M1) was performed. (**B**) Preservation of alveolar ridge and soft tissue at M1 site (circled with blue dashed line) two weeks after decoronation. Red arrows indicate some roots are exposed through the soft tissue. (**C**) Semilunar flap incision at M1 site. (**D**) Extraction of M1 roots. (**E**) M1 socket after complete removal of all five roots. (**F**) A standardized alveolar bone defect (bone defect measuring approximately 3 mm in diameter and 2 mm depth) was drilled using low-speed tungsten carbide round bur (diameter 2.7 mm) by aiming the center of inter-radicular septa till the depth that the bur head just submerged into the level of soft tissue surface. (**G**) The bone defects were randomly assigned to be filled with metformin-incorporated gelatin/nano-hydroxyapatite sponge (GHMS), Sinbone granules (60%HA + 40%β-TCP), or Bio-Oss Collagen^®^ (**H**). Primary closure was achieved using resorbable vicryl 6-0 sutures. (**I**) Micro-computed tomography assessments of alveolar bone dimension at maxillary first molar (M1) sites, namely (1) distal buccal bone height, (2) distal palatal bone height, (3) distal middle buccal bone height, (4) middle palatal bone height, (5) middle graft height, (6) mesial buccal bone height, (7) mesial palatal bone height, (8) middle maximum alveolar bone width, and (9) 3D bone volume. Three-dimensional (3D) bone volume was calculated by adding up consecutive surfaces of interest on the transaxial sections mesiodistally throughout the M1 site excluding the graft material on the grafting/experimental side (images on the left, indicated by pink arrow). On the non-extraction (control) side, bone contour volume was calculated by adding up consecutive surfaces of interest on the transaxial sections including the roots (images on the right, indicated by blue arrow).

All procedures complied with protocols pre-approved by the Institutional Animal Care and Use Committee of National Taiwan University IACUC#20180197). Seventy-two eight-week-old male Wistar rats with average weight of 350 g were randomly assigned to receive extraction-only or grafting at the maxillary first molar (M1) site on one side (experimental side) and the contralateral maxillary M1 served as unmanipulated (non-extraction) control (UCtrl). Rats were anesthetized using 1:2 concentrations of Zoletil and Rompum as previously described [44] with local anesthesia (0.1 mL, 2% lidocaine with 1:100,000 epinephrine) infiltration adjacent to M1 on the experimental side. For the extraction group, M1 was luxated until significant elevation and then extracted using a dental explorer without further enlargement of the extraction sockets. For the grafting groups, decoronation of M1 was first performed using a tungsten carbide round bur (ISO 027, diameter 2.7 mm) with a low-speed dental handpiece (1000 rpm; SM, Osstem, Taiwan) under continuous saline irrigation by removing the crown and perforating the furcation until separation of the roots. After two weeks of secondary soft tissue healing, a semilunar incision was made to expose the alveolar crest of M1 and the remaining roots of M1 were completely removed under the same anesthetic procedure. A standardized critical size alveolar bone defect was created using low-speed tungsten carbide round bur (diameter 2.7 mm) by aiming the center of interradicular septa until the depth that the bur head was just submerged into the soft tissue surface. The bone defects were randomly assigned to be filled with gelatin/nHA/metformin scaffold/sponge (GHMS), Sinbone granules (60%HA + 40%β-TCP; Purzer Pharmaceutical Co., Ltd., Taipei, Taiwan), or Bio-Oss Collagen^®^ (Geistlich Biomaterials, Wolhusen, Switzerland). Primary closure was achieved using resorbable vicryl 6-0 sutures (Figure 6A–H). The rats were euthanized by CO_2_ asphyxiation and decapitated at designated time points (4, 8, or 12 weeks) after the extraction/grafting procedure (six rats per group per time point). The skull was fixed in 10% formalin for micro-computed tomography (micro-CT) and histological examinations.

#### 4.5.1. Micro-Computed Tomography (Micro-CT) Analysis

The rat skulls were scanned with high-resolution micro-CT (Skyscan1076, Kontich, Belgium) at a pixel size of 18 µm. Ten parameters, namely distal buccal bone height, distal palatal bone height, middle buccal bone height, middle palatal bone height, middle graft height, mesial buccal bone height, mesial palatal bone height, middle maximum bone width, three-dimensional (3D) bone (contour) volume, and bone volume density (BV/TV; ratio of bone volume to total volume) were measured at the M1 site (Figure 6I). For the measurement BV/TV, a semicircular region of interest (ROI) was accurately positioned over the M1 defect (4 × 1 × 1 mm), ensuring that the surrounding alveolar bone was covered. DataViewer and CTAn SkyScan software were used for analyses of alveolar bone regeneration.

#### 4.5.2. Histological Analysis

After micro-CT scanning, the specimens were fixed, decalcified, and embedded in paraffin. The paraffin-embedded specimens were sectioned at a thickness of 5 μm, stained with hematoxylin and eosin (H&E) or Goldner’s trichrome, and viewed under a light microscope (IX71; Olympus, Tokyo, Japan).

### 4.6. Statistical Analysis

Data were expressed as mean ± standard deviation (SD). Statistical analysis was performed using one-way ANOVA with Bonferroni post hoc comparisons; the significance level was set at *p*-value < 0.05.

## 5. Conclusions

In a critical size rat alveolar bone defect, we have demonstrated the superior performance of metformin-loaded gelatin/nHA sponge (GHMS) in bone regeneration compared with extraction-only, calcium phosphate–only (Sinbone), and calcium phosphate plus collagen (Bio-Oss Collagen^®^) control groups. Micro-CT showed preservation of the alveolar ridge dimensions and an increase in bone volume and bone fill in the GHMS group. Histological staining showed an increased number of osteocytes, presence of Haversian canals in the newly formed bone, and more bone formation with a lower percentage of connective tissue and residual scaffold at the defect sites grafted with GHMS. In vitro results showed that GHMS promoted osteogenic differentiation of human mesenchymal stem cells. These excellent results may be attributed to the greater biocompatibility, relatively fast degradation of the scaffold, large pores that allow vascularization, and higher bioactivity properties of the components of the scaffold (gelatin, nHA, and metformin) that favor direct osteogenesis. This GHMS may be easily reshaped and adapted to the size and location of the alveolar bone defect. It shows sufficient mechanical strength to prevent soft tissue collapsing. These findings demonstrate that GHMS may be used as a potential bone substitute to effectively regenerate alveolar bone formation. In the future, further investigations are required in order to examine the optimal metformin concentration in a controlled delivery system for bone healing in large bone defects and to test GHMS in chronically infected defect model.

## Figures and Tables

**Table 1:** Functional groups and mode of vibration from FTIR spectra of the GHMS.

Peak Position on FTIR Spectra (cm^−1^)	Assignment of Bonds	Mode of Vibration
3420	O-H	stretching
3369	N-H	primary stretching vibration
3294	N-H	primary stretching vibration
3155	N-H	secondary stretching
2800–2950	C-H	stretching
1626	C-N	stretching
1567	C-N	stretching
1063	(PO4)3-	stretching

## Data Availability

The datasets used and/or analyzed in the current study are available from the corresponding author on reasonable request.
